# Production of Mare Chorionic Girdle Organoids That Secrete Equine Chorionic Gonadotropin

**DOI:** 10.3390/ijms24119538

**Published:** 2023-05-31

**Authors:** Riley E. Thompson, Mindy A. Meyers, Jennifer Palmer, D. N. Rao Veeramachaneni, Christianne Magee, Amanda M. de Mestre, Douglas F. Antczak, Fiona K. Hollinshead

**Affiliations:** 1 Department of Clinical Sciences, Colorado State University, Fort Collins, CO 80523, USA; 2 Animal Reproduction and Biotechnology Laboratory, Colorado State University, Fort Collins, CO 80523, USA; 3 Department of Biomedical Sciences, Colorado State University, Fort Collins, CO 80523, USA; 4 Baker Institute for Animal Health, College of Veterinary Medicine, Cornell University, Ithaca, NY 14853, USA

**Keywords:** chorionic girdle, trophoblast, endometrial cup, mare, equine, horse, organoid, in vitro, cell culture, 3D

## Abstract

The equine chorionic girdle is comprised of specialized invasive trophoblast cells that begin formation approximately 25 days after ovulation (day 0) and invade the endometrium to become endometrial cups. These specialized trophoblast cells transition from uninucleate to differentiated binucleate trophoblast cells that secrete the glycoprotein hormone equine chorionic gonadotropin (eCG; formerly known as pregnant mare serum gonadotropin or PMSG). This eCG has LH-like activity in the horse but variable LH- and FSH-like activity in other species and has been utilized for these properties both in vivo and in vitro. To produce eCG commercially, large volumes of whole blood must be collected from pregnant mares, which negatively impacts equine welfare due to repeated blood collections and the birth of an unwanted foal. Attempts to produce eCG in vitro using long-term culture of chorionic girdle explants have not been successful beyond 180 days, with peak eCG production at 30 days of culture. Organoids are three-dimensional cell clusters that self-organize and can remain genetically and phenotypically stable throughout long-term culture (i.e., months). Human trophoblast organoids have been reported to successfully produce human chorionic gonadotropin (hCG) and proliferate long-term (>1 year). The objective of this study was to evaluate whether organoids derived from equine chorionic girdle maintain physiological functionality. Here we show generation of chorionic girdle organoids for the first time and demonstrate in vitro production of eCG for up to 6 weeks in culture. Therefore, equine chorionic girdle organoids provide a physiologically representative 3D in vitro model for chorionic girdle development of early equine pregnancy.

## 1. Introduction

The equine chorionic girdle is the precursor tissue to endometrial cups that secrete the glycoprotein hormone equine chorionic gonadotropin (eCG), also known as pregnant mare serum gonadotropin (PMSG). The chorionic girdle is a unique placental structure in the mare that begins formation 25 to 35 days after ovulation (day 0) and is located at the junction between the growing allantois and regressing yolk sac [1]. The specialized trophoblast cells of the chorionic girdle begin to differentiate from uninucleate to binucleate cells as early as day 31 and increase in number until day 35 [2]. These terminally differentiated binucleate trophoblast cells of the chorionic girdle invade the endometrium from days 36 to 38 to form unique ‘endometrial cups’ that are the source of eCG. By day 40 eCG can be detected in maternal circulation. The eCG peaks in concentration at about day 70 and circulates until about day 120 of gestation [3,4,5]. Endometrial cups persist until day 100–120 of gestation when they ultimately slough from the endometrial surface [6]. Due to its luteinizing hormone (LH)-like properties, eCG is an essential hormone for pregnancy maintenance in the mare. These properties result in direct luteotropic effects by eCG on the primary corpus luteum and formation of supplementary corpora lutea (CLs) that will increase progesterone production to preserve a uterine environment conducive to pregnancy maintenance [6,7].

In addition to its importance in pregnancy maintenance in the mare, eCG administration is essential for many domestic and non-domestic animal breeding programs, which include cattle, sheep, goats, pigs, cats, and captive wildlife species [8,9,10,11]. LH, follicle stimulating hormone (FSH), and eCG are all glycoprotein hormones which are heterodimers with a common alpha subunit and hormone-specific beta subunit peptide chains [12]. Equine LH (eLH) and eCG have identical beta subunits because they are produced by the same gene [13,14,15]; thus, these hormones have similar biological activity in the mare. However, eLH and eCG have different post-translational glycosylation patterns, which is primarily why eCG has a much longer half-life than eLH [15,16]. The mechanism of action for “FSH-like” activity of eCG when administered to non-equid species is not fully elucidated but is believed to be due to structural similarities between eCG and FSH of those non-equids [15]. As eCG can have both FSH- and LH-like activity, it can be used to induce both estrus and ovulation depending on species and dose rate [10,17]. To produce eCG for commercial use, large volumes (up to 5 L) of whole blood are collected from mares (days 40–120 gestation) once or twice weekly to coincide with eCG secretion [17]. This traditional in vivo method of producing eCG commercially has significant welfare ramifications for the horses used in these programs, specifically for the aforementioned reasons and because mares often are not well habituated to people, which can lead to human and animal injuries [17]. Furthermore, these operations have unwanted foals as a by-product, which leads to an excess in the horse population. For the above reasons, commercial availability of eCG has become limited. As a solution, several groups have attempted to produce eCG in vitro, but no one has yet achieved a commercial in vitro system that can produce eCG in sufficient quantities long-term [18,19,20].

Organoids are three-dimensional (3D) cell clusters generated from stem or organ progenitor cells that self-organize and can remain both genetically and phenotypically stable throughout long-term culture (months to >1 year, depending on the tissue of origin) [21,22,23]. Organoids recapitulate key physiological aspects of the organ of origin and have become an alternative in vitro model that mimics normal physiology long-term [24]. Organoids have been produced from many types of tissues in a variety of species and extensively studied in human liver, prostate, brain, endometrium, and trophoblast organoid culture systems [25,26,27,28,29,30,31,32]. Organoids derived from mare endometrium and oviductal tissue have also been reported [31,32,33]. In 2018, human trophoblast organoids were reported to grow long-term (>1 year) and to produce human chorionic gonadotropin (hCG) [30], which is a glycoprotein hormone with similar properties to eCG. Here we report the generation of organoids derived from equine chorionic girdle cells that produce eCG in vitro. This data provides an improved in vitro model system for studying chorionic girdle development of early pregnancy in the mare and may potentially result in an in vitro source of eCG production.

## 2. Results

### 2.1. Preliminary Data

Preliminary data was performed utilizing the same culture conditions as Trial 1 with the exception of using culture medium that has been reported for production of equine endometrial organoids [31,32] rather than the trophoblast organoid medium that was implemented for the other trials reported here. Chorionic girdle organoid culture with endometrial organoid medium resulted in organoid growth and proliferation over a 2 week period, including 1 passage. These organoids produced eCG in spent medium during passage 0 (P0; 242.3 ng/mL) that declined rapidly during P1 (6.5 ng/mL).

### 2.2. Trial 1

**Figure 1:**
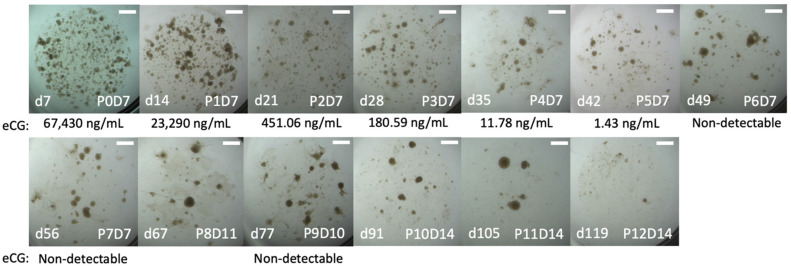
Brightfield images with corresponding eCG for chorionic girdle organoids in Trial 1. Brightfield images of chorionic girdle organoids for Trial 1 with labels for passage (P) and day (D) and for total days (d) that image was captured and the corresponding concentration of equine luteinizing hormone (eLH), as a proxy for equine chorionic gonadotropin (eCG), from spent culture medium. Scale bar = 1 mm.

**Figure 2:**
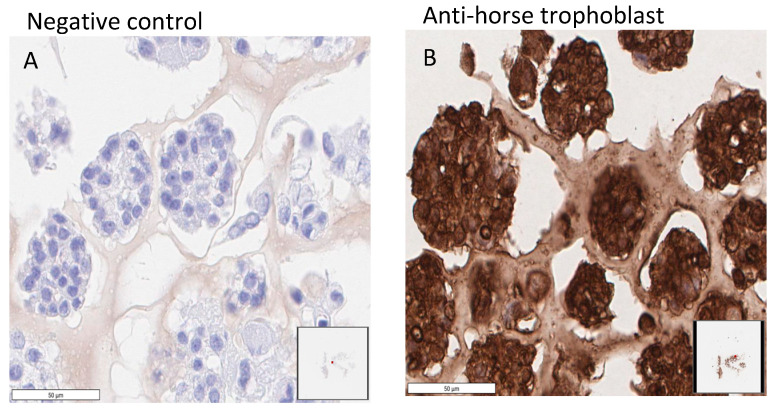
Immunohistochemical staining for chorionic girdle organoids. Organoids at passage 0, day 7 (P0D7) from Trial 1 displaying negative control (**A**) and positive staining for anti-horse trophoblast antibody (**B**). Scale bar = 50 μm.

In Trial 1 (see Table 1), organoid phenotype, as described for human trophoblast organoids [30], was maintained for 17 weeks through 12 passages (Figure 1). Culture was ceased when organoids failed to continue to proliferate. Chorionic girdle organoids in this trial produced high levels of eCG for 4 weeks (P0: 67,430 ng/mL; P1: 23,290 ng/mL; P2: 451 ng/mL; and P3: 181 ng/mL LH) before a substantial decline at and beyond the fourth passage (12 ng/mL and lower). Confirmation of the organoid origin was performed utilizing IHC, in which an anti-horse trophoblast antibody (102.1) displayed specific staining in all organoid cells from P0D7 (Figure 2).

### 2.3. Trial 2

**Figure 3:**
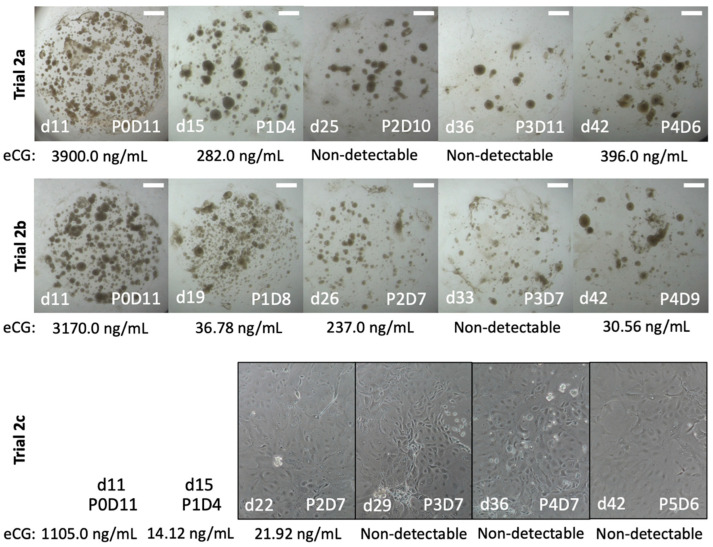
Brightfield images with corresponding eCG for chorionic girdle organoids in Trial 2. Brightfield images of chorionic girdle organoids (Trials 2a, 2b) and monolayer (Trial 2c) with labels for passage (P) and day (D) and for total days (d) that image was captured and the corresponding concentration of equine luteinizing hormone (eLH), as a proxy for equine chorionic gonadotropin (eCG), from spent culture medium. All treatments were cultured for 6 weeks total. Scale bar = 1 mm.

**Figure 4:**
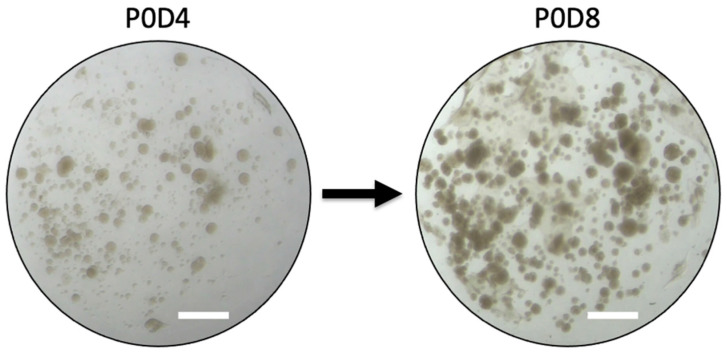
Brightfield images demonstrating organoid growth and proliferation within a passage in a single well. Brightfield images of chorionic girdle organoids (Trial 2b) from a single well with labels for passage (P) and day (D) that demonstrate growth and proliferation of organoids within a passage. Scale bar = 1 mm.

In Trial 2, all three treatments (a, b, and c; see Table 1) were cultured for 6 weeks with varying times for passage based on organoid growth (Figure 3 and Figure 4). Chorionic girdle organoids from Trial 2a secreted high levels of eCG for the first two passages (P0: 3900 ng/mL; P1: 282 ng/mL) then was non-detectable until organoids were plated at a more concentrated rate for the final passage (P4: 396 ng/mL). In Trial 2b, organoids secreted higher levels of eCG for the first 3 passages (P0: 3170 ng/mL; P1: 37 ng/mL; P2: 237 ng/mL) then decreased to non-detectable levels until they again were pooled for the final passage (P4: 31 ng/mL). In Trial 2c, the corresponding monolayer cell cultures initially displayed high levels of eCG at P0 (1105 ng/mL) before rapidly decreasing (P1: 14 ng/mL; P2: 22 ng/mL) and reached non-detectable levels at P3 that persisted until culture was discontinued after P5.

**Figure 5:**
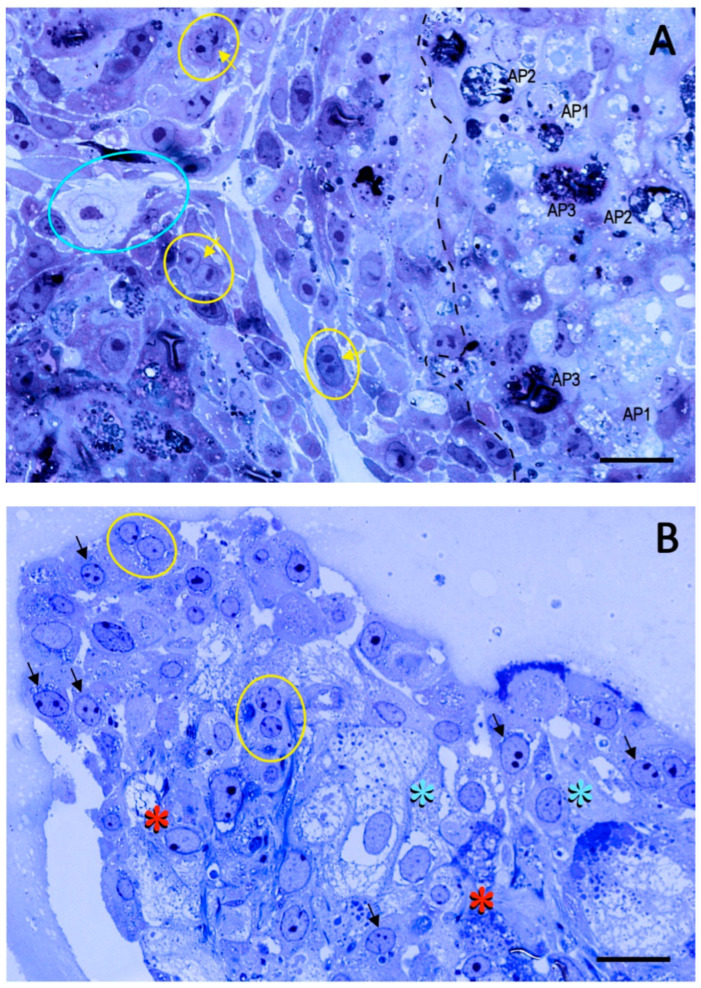
Light micrographs of toluidine blue stained, 1-μm-thick sections of equine chorionic girdle organoids from Trial 2a at two timepoints ((**A**): P1D3, (**B**): P4D6). Scale bars = 20 μm. (**A**) Portions of three organoids are seen. As the girdle cells (blue circle) differentiate into binucleate trophoblast cells they manifest a series of progressive morphological changes, beginning from nascent uninucleate trophoblast cells to transitioning trophoblast cells with a nucleus containing two nucleoli (yellow circles with arrows), developing a bifurcation of the nucleus, and finally becoming a binucleate trophoblast cell (yellow circles seen in Panel (**B**)), characteristic of invasive trophoblast cells that are associated with endometrial cups. In the organoid on the right, different sizes of autophagosomes (AP) are seen in the cells undergoing autophagy; these are sequentially numbered as AP1 (early), AP2, and AP3 (late) as degradation of cellular organelles such as endoplasmic reticulum, mitochondria, and ribosomes progresses and coalesce. Due to the conspicuous detrimental changes in this tissue aggregate (enclosed by dashed line), the individual cellular margins are not discernible. (**B**) In this organoid, several transitioning trophoblast cells with a nucleus containing two nucleoli (black arrows) and fully differentiated binucleate trophoblast cells (yellow circles) are seen, indicating that the organoids maintain necessary structures required for functionality (i.e., eCG secretion) at day 42 in culture. That girdle cells also are active at this stage of culture is evident from apparent foci of autophagosomes (red asterisks) and vacuolizations (teal asterisks).

**Figure 6:**
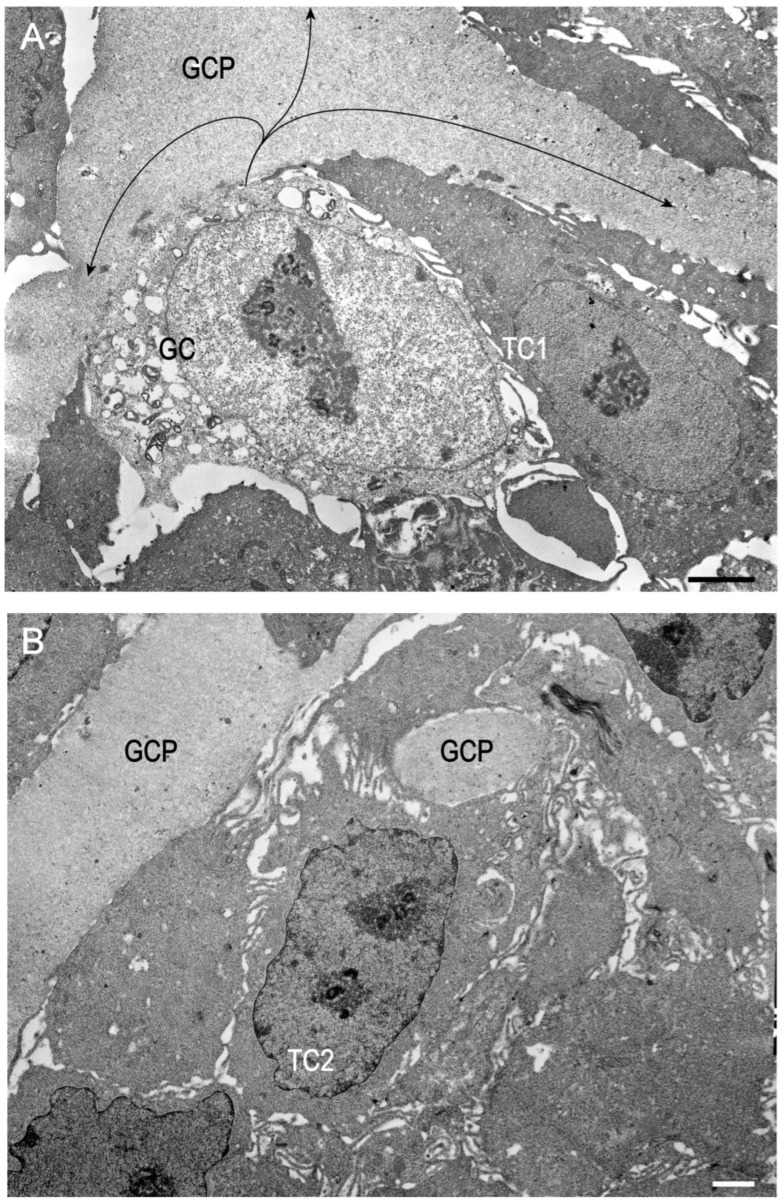

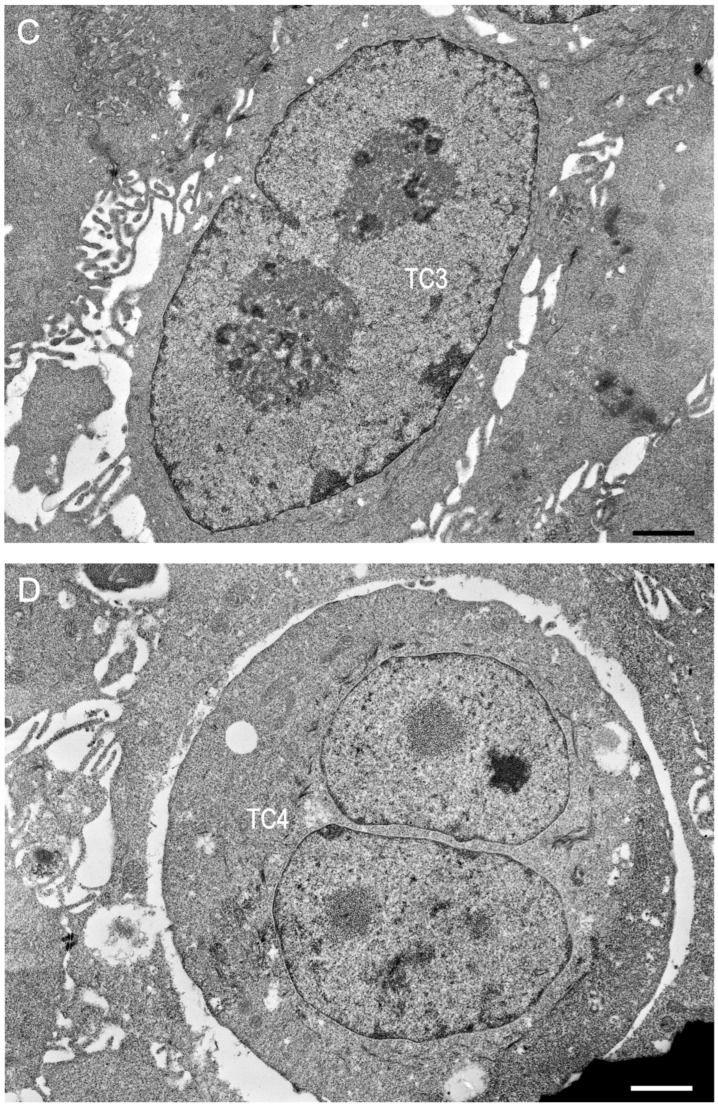
Transmission electron micrographs of equine chorionic girdle organoids from Trial 2a (P1D3, 14 days in culture). Scale bars = 1 μm. The number designation in each trophoblast cell (TC) indicates its sequential progression of development following its differentiation from uninucleate trophoblast cell. The description of the developmental events in organoids in this sample is based on the classical work on the origin of equine endometrial cups by Allen et al. [4]. (**A**) A girdle cell (GC) with its process (GCP; indicated by arrow) pervading the interstitium is seen. A nascent uninucleate trophoblast cell, TC1, is seen adjacent to the girdle cell. The nuclei in both GC and TC1 manifest euchromatin indicating that both cells are active. The arrows indicate GCP location. (**B**) Note that the nucleus of transitioning trophoblast cell, TC2, has two nucleoli in its nucleus. Note also that the pervading girdle cell process (GCP) penetrates and enters interstitium of the organoid. (**C**) Note the indentation in the nuclear membrane of this trophoblast cell marking the beginning of bifurcation of this transitional trophoblast cell, TC3. (**D**) Bifurcation of the nucleus of TC3 is complete, resulting in the hallmark binucleate trophoblast cell, TC4. These terminally differentiated binucleate trophoblast cells are the source of equine chorionic gonadotropin (eCG), also known as pregnant mare serum gonadotropin (PMSG).

The structural integrity of the chorionic girdle organoids (Trial 2a) was evaluated by light microscopy at P1D3 and P4D6, and by TEM at P1D3. Thick sections stained with toluidine blue (Figure 5) demonstrated the presence of binucleate trophoblast cells in both P1 and P4 organoids. TEM images of approximately ten organoids reveal salient features of chorionic girdle cells, such as girdle cell processes [4], and their final differentiation into binucleate cells (Figure 6). The girdle cells are shown to differentiate through a series of progressive morphological changes, beginning from uninucleate trophoblast cells to transitioning trophoblast cells with a nucleus containing two nucleoli, developing a bifurcation of the nucleus, and finally becoming a binucleate trophoblast cell, which is characteristic of trophoblast cells that are associated with endometrial cups. Autophagosomes are seen in some of the cells undergoing autophagy. Cells comprising the organoids are undergoing both autophagy and are actively dividing.

## 3. Discussion

Culture of equine chorionic girdle cells in vitro was first reported in 1972 [1], wherein pieces of equine chorionic girdle collected at Day 35 after ovulation were cultured as explants (M199 media with 20% fetal calf serum and 50 mg insulin, 50 mg ascorbic acid, 50 units penicillin, and 50 units streptomycin per mL at 37 °C, 5% CO_2_), but were observed to attach to the dish and gain confluence like a monolayer-type cell culture [1]. These initial chorionic girdle cultures produced over 400 IU eCG (equivalent to ~1972 ng/mL eCG) within 30 days of culture, peaked between days 40–60 of culture, and declined steadily until day 180 of culture [1]. This first report still remains the most successful published attempt to culture chorionic girdle in vitro, but differentiation of chorionic girdle cells in vitro with long-term eCG production remains challenging. In a more recent study, equine chorionic girdle cells collected from Day 34 conceptuses were cultured in a dish, either with or without 10% fetal calf serum. The chorionic girdle cells cultured with serum adhered to the culture flask as a monolayer, but the cells cultured without serum formed non-adherent spherical clumps, referred to as ‘vesicles’ by the authors [34]. These ‘vesicles’ would be referred to as ‘spheroids’ using current terminology, and were cultured for up to 17 days with eCG evaluation of spent medium performed after 3 days in culture. While this report represented a critical breakthrough by maintaining chorionic girdle in a non-explant 3D cell culture, a longer culture period with the maintenance of physiologically functioning chorionic girdle cells is necessary to evaluate chorionic girdle development and function effectively in vitro. Attempts to manufacture synthetic alternatives to eCG have not been successful [17,35] until recently; production of a recombinant eCG utilizing a lentivirus vector has been described that resulted in biological activity in cattle [36]. These results offer promise as an alternative to in vivo-produced eCG.

In the present study, a protocol describing organoid generation from first trimester human trophoblast tissue [30] was adapted for the culture of equine chorionic girdle cells. The human trophoblast-derived organoids differentiated into both syncytiotrophoblast and extravillous trophoblast cells and were maintained in culture for over a year while retaining genetic stability. Furthermore, the syncytiotrophoblasts in that study secreted the placenta-specific human chorionic gonadotropin (hCG). In the present study, equine chorionic girdle organoids also demonstrated chorionic gonadotropin production, illustrating organoid functionality.

The trials in this study were designed based on tissue availability and progressed as additional knowledge was generated throughout the study. Thus, preliminary experiments utilized organoid culture medium that had been successful for other equine reproductive tissues [31,33] but was found to be unsuccessful for eCG production beyond P0. Trial 1 utilized trophoblast organoid medium, as reported for use in human trophoblast organoid generation [30], and was found to be successful for production of eCG for at least 4 weeks of chorionic girdle organoid culture. Trial 2 then replicated the conditions of Trial 1 but only 6 weeks of culture were utilized, as 4 weeks appeared to be the extent of eCG production by the organoids demonstrated in Trial 1. Furthermore, methods of chorionic girdle cell isolation (enzymatic vs. mechanical) and incubator conditions (5% vs. 8% CO_2_) were compared (Trial 2a vs. 2b) to determine if one of these methods improved organoid viability and function, as described in another chorionic girdle culture publication [34]. Trial 2 also compared organoids to traditional monolayer cell culture to demonstrate the disparity between the two cell culture systems for cellular function (i.e., eCG production).

Secretion of eCG would not be possible without the presence of binucleate trophoblast cells of the chorionic girdle. As binucleate cells are the hallmark of the invasive trophoblast cells of the equine chorionic girdle, the presence of this cellular structure was also assessed to provide a better understanding of the relationship between the morphologic characteristics of chorionic girdle and the in vitro production of eCG. Toluidine blue and TEM images from P1D3 and P4D6 (Trial 2a) demonstrated the presence of binucleate cells in the chorionic girdle organoids. Furthermore, immunohistochemistry utilizing an anti-horse trophoblast antibody confirmed that the organoids were comprised of only equine trophoblast cells. Additionally, girdle cell processes were present (Figure 6), which were first described by Allen et al. [4], after performing TEM imaging of chorionic girdle tissue collected from pony mares on days 36–38. Moreover, organoids demonstrated both cellular divisions, confirmed by growth and proliferation of organoids within a passage (Figure 4), and differentiation from uninucleate to binucleate trophoblast cells, which was demonstrated by TEM (Figure 6); this combination of features has not been reported previously in in vitro equine chorionic girdle cell culture. The presence of all the above characteristics indicates that chorionic girdle organoids display key features of equine chorionic girdle tissue in vivo, which creates a biomimetic environment to model the chorionic girdle development of early pregnancy in the mare.

Preliminary experiments began by utilizing equine endometrial organoid medium [31], which resulted in an immediate decrease in eCG secretion after P0. As a result, culture medium was changed to trophoblast organoid medium [30] for the following trials. Both of these media contain similar supplements with a few distinct differences. The equine endometrial medium contained penicillin/streptomycin, insulin-transferrin-selenium, nicotinamide, FGF10, noggin, and SB202190, while the human trophoblast medium contained primocin, CHIR99021, Rspondin-1, FGF2, HGF, Y27632, and prostaglandin E_2_. Given the difference in eCG production between the two trials, it is presumed that these differences in supplements impacted eCG production by the organoids. Although beyond the scope of this study, an elimination trial is needed to determine which supplements impaired and/or increased eCG production.

In Trials 2a and 2b, eCG declined to nondetectable levels before increasing to detectable levels again (Figure 3). The authors attribute this finding to having increased the seeding density of organoids per well toward the end of the culture period. This was attempted because anecdotal evidence has indicated that higher seeding densities may improve organoid growth.

Some of the limitations of this study include the limited number of replicates performed due to the challenging nature of collecting this tissue. Due to the limited number of samples, statistical analysis comparing eCG production among treatments was unable to be performed. Therefore, the differences in eCG production among treatments is considered descriptive data for the development of this model of the chorionic girdle in early equine pregnancy. Another limitation is a definable number of cells was not plated per well. This was because anecdotal evidence provided by other researchers who work with chorionic girdle tissue indicated that dissociating tissues to single cells is often detrimental to cellular viability. Therefore, to optimize the likelihood of success, we did not dissociate to single cells before plating in UltiMatrix™ droplets. Due to this limitation, ensuring that the same number of cells were plated per well in each treatment was not possible; therefore, quantifying the concentration of eCG per plated cell was not feasible. Thus, some of the variation in eCG concentration among trials and treatments may be due to variation in number of cells plated per well rather than an effect of treatment.

This is the first report of the generation of organoids derived from equine chorionic girdle cells. Furthermore, eCG production by the organoids persisted for 4 to 6 weeks, which is longer than what is reported with the monolayer cell cultures in the present study. Future research will involve trialing additional supplements to determine whether these may increase organoid proliferation and/or prolong in vitro production of eCG. In addition to modification of organoid culture medium supplements, implementation of a bioreactor culture system may also improve organoid longevity by creating continuous movement of culture medium to deliver nutrients and gases and remove waste surrounding the organoids. Lastly, developing a cryopreservation protocol that allows freezing/thawing of chorionic girdle tissue without affecting its viability or functional capacity would reduce the challenges associated with obtaining this tissue. In summary, equine chorionic girdle organoids offer an alternative in vitro model system to study physiology, pathology, and disease therapeutics for early pregnancy in the mare, and with further optimization, equine chorionic girdle organoids may provide an in vitro source of eCG to address animal welfare ramifications and reduced access associated with currently available commercially produced eCG.

## 4. Materials and Methods

### 4.1. Animals

All animal procedures were approved by the Colorado State University (CSU) Institutional Animal Care and Use Committee IACUC (protocol #979). Fetuses and their extra-fetal membranes (n = 3) were recovered by flushing the mare’s uterus at Day 33 or 34 post-ovulation, as previously described [37,38].

### 4.2. Experimental Design

Unless stated otherwise, all chemicals were purchased from Sigma-Aldrich Chemicals, St. Louis, MO, USA.

The first chorionic girdle that was collected was used to assess culture medium conditions by utilizing medium reported for equine endometrial organoids [31]. Results of the preliminary experiment indicated that eCG production declined rapidly under these culture conditions. Following this preliminary experiment, two trials with the remaining two chorionic girdle tissues were performed (Table 1). For Trial 1, chorionic girdle tissue was digested enzymatically with collagenase V (0.4 mg/mL) and dispase II (1.25 U/mL) in RPMI-1640 medium, then cultured using medium that was previously reported to support growth of organoids derived from human trophoblast [30], and placed in 37 °C, 5% CO_2_. Trial 2 used chorionic girdle tissue collected from one mare that was split into three treatments. Trial 2a utilized the same digestion and culture conditions as Trial 1. In Trial 2b, chorionic girdle tissue was scraped with a scalpel blade to mechanically isolate the cells without enzymatic digestion, then cultured using medium that was previously reported to support growth of organoids derived from human trophoblast [30], and placed in 37 °C, 8% CO_2_. In Trial 2c, chorionic girdle tissue was scraped similar to Trail 2b, but cultured as a monolayer using medium that was previously reported to support growth of equine chorionic girdle monolayers [34], and placed in 37 °C, 8% CO_2_.

### 4.3. Organoid Culture

**Figure 7:**
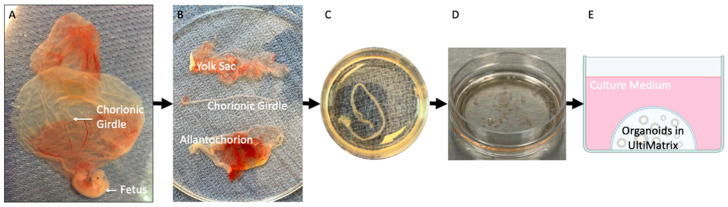
Depiction of chorionic girdle isolation for organoid culture. Images of (**A**) intact Day 33 equine fetus and fetal membranes, (**B**) chorionic girdle dissected from fetal membranes, (**C**) isolated chorionic girdle, and (**D**) 1 mm^3^ pieces of chorionic girdle for digestion. (**E**) Diagram of a single well of organoids embedded in the extracellular matrix UltiMatrix™ and overlaid with culture medium.

Conceptus tissues were transported to the laboratory for isolation in phosphate-buffered saline (PBS without Ca^++^ and Mg^++^) within 10 min of collection. The chorionic girdle was mechanically isolated from the surrounding fetal membranes using surgical scissors and light microscopy (Figure 7). Cells were released either by cutting into 1–2 mm^3^ pieces followed by 5 min of enzymatic digestion at 37 °C in collagenase V (0.4 mg/mL) and dispase II (1.25 U/mL) in RPMI-1640 medium, or mechanically isolated by being scraped from the basement membrane (see Table 1). Following cell isolation, a 25 μL droplet of UltiMatrix™ Reduced Growth Factor Basement Membrane Extract (Cultrex™) containing chorionic girdle cells was placed in each well of a 48-well plate. After 30 min at 37 °C, UltiMatrix™ droplets were overlaid with human trophoblast organoid medium [TOM: DMEM/F12 without phenol red, 2.5 mM L-glutamine, 1% N2 (Thermo Fisher; Waltham, MA, USA), 2% B27 supplement minus vitamin A (Thermo Fisher), 100 mg/mL Primocin (Invivogen; San Diego, CA, USA), 1.25 mM N-acetyl-L-cysteine (Millipore Sigma; Burlington, MA, USA), 0.5 mM TGFβ/Alk inhibitor A83-01 (Tocris Bioscience; Minneapolis, MN, USA), 1.5 mM CHIR99021 (Tocris), 50 ng/mL recombinant human EGF (R&D Systems; Minneapolis, MN, USA), 80 ng/mL recombinant human Rspondin-1 (R&D Systems), 100 ng/mL recombinant human FGF2 (Peprotech; Cranbury, NJ, USA), 50 ng/mL recombinant human HGF (Peprotech), 5 mM Y-27632 (EMD Millipore; Burlington, MA, USA), 2.5 mM prostaglandin E_2_] [30]. Cells were maintained at 37 °C, 5% or 8% CO_2_ (see Table 1). Half of the organoid medium was removed and replaced every 2–3 days. Passaging was performed every 7–14 days, contingent upon growth and gross viability (light vs. dark color) of organoids.

To passage, culture medium was removed from each well and replaced with 250 μL DMEM/F12. The contents of the wells were transferred to microcentrifuge tubes and centrifuged at 600× *g* for 6 min. The resulting pellet was dissociated by repeated manual pipetting (500×) and then washed in DMEM/F12 through centrifugation. The resulting pellet was again dissociated by manual pipetting (300×), washed in DMEM/F12 through centrifugation, and the dissociated cells plated, as described above. Initially, the passage rate of organoids was performed at a rate of 1 to 4 (one well was dissociated for plating into 4 wells after passage), but this was reduced to 1 to 1 with the necessary mechanical dissociation, which breaks apart the organoid cell clusters to prevent cellular death in the center of the organoids and allows additional cellular proliferation, in the final passages of the culture period.

### 4.4. Monolayer Cell Culture

Fetal tissues were transported and isolated as above (see Organoid Culture). Cells were scraped from the basement membrane in Handling Medium [HM; MEM with Earle’s Salts, 25 mM HEPES, 100 U/mL Penicillin, 0.1 mg/mL Streptomycin, 0.1 mM Pyruvate, 2 mM Glutamax, 5% Bovine Serum Albumin] [39,40] and then centrifuged at 600× *g* for 6 min. The resulting pellet was resuspended in equine chorionic girdle monolayer medium [Monolayer medium: DMEM/F12 without phenol red with 2.5 mM L-glutamine, 100 U/mL penicillin-streptomycin, 10% (*v*/*v*) FBS] [34] and plated at 250 mL per well in a 48-well plate. Culture medium was replaced every 2–3 days.

To passage, culture medium was removed from each well and the cells were washed with 250 μL pre-warmed PBS. Then, 100 μL pre-warmed (37 °C) 0.25% trypsin/0.5 mM EDTA (Corning) was added for 2 min while gently scraping the well with a pipette tip to release cells from the bottom of the well. Subsequently, 100 μL monolayer medium was added to each well, transferred to microcentrifuge tubes, and centrifuged at 600× *g* for 5 min. The cell pellet was resuspended in monolayer medium and plated, as described above.

### 4.5. Immunohistochemistry (IHC)

Organoids [Trial 1; passage 0, day 7 (P0D7)] were soaked in Organoid Harvesting Solution (Cultrex™), a non-enzymatic solution that depolymerizes UltiMatrix™, and washed in PBS through centrifugation [20]. Organoid pellets were fixed in 4% paraformaldehyde for 30 min before embedding in 2% agarose gel [31]. The organoids in agarose gel were stored in 70% ethanol until routine embedding. Tissue sections embedded in paraffin wax were mounted on Superfrost Plus™ slides. Slides were deparaffinized then rehydrated through graded alcohols to distilled water. Then, antigen retrieval with citrate buffer (pH 6; 90 °C for 20 min) was performed. The slides were then blocked with hydrogen peroxide and washed in distilled water. Next, the slides were incubated with the monoclonal primary antibody (102.1, anti-horse trophoblast [34,41]; diluted 1:100) and biotin conjugated mouse IgG (Vector; #BA-2000), followed by horseradish peroxidase (HRP)-conjugated streptavidin (Vector; #SA5004). A negative control utilized the same procedure without the primary antibody incubation step. Lastly, color was developed using 3,3′-Diaminobenzidine (DAB) and counterstained with hematoxylin. All incubations were performed at room temperature with Tris-buffered saline, 0.1% Tween^®^ 20 detergent (TBST) as a washing buffer. The slides were evaluated for location and intensity of staining.

As an additional control, the organoids also were processed using frozen tissue sections, as previous publications reporting the primary antibody (102.1) utilized frozen tissue sections [34,41]. Briefly, organoid pellets were fixed in 4% paraformaldehyde for 5 min then stored in 15% sucrose solution until processing. For processing, they were placed in optimal cutting temperature compound (O.C.T.; Thermo Fisher) cryo-embedding medium, frozen in liquid nitrogen, and stored at −80 °C until routine cryo-sectioning. Staining was similar to paraffin-embedded organoids.

### 4.6. Transmission Electron Microscopy (TEM)

A sample of organoids (Trial 2a, P1D3) was soaked in Organoid Harvesting Solution (Cultrex™) to remove UltiMatrix™ and washed in PBS through centrifugation [20]. Organoid pellets were fixed in 4% glutaraldehyde with 5% sucrose in 0.1 M sodium cacodylate (pH 7.4) for two hours, stored in 0.1 M cacodylate buffer at 4 °C, and processed for both light and transmission electron microscopy, as previously described [33], to characterize ultrastructural features of the cellular constituents of the chorionic girdle organoids, with attention to the presence of characteristic binucleate cells.

A sample from the same trial (Trial 2a) at a later time period (P4D6) was processed similar to the P1D3 sample to assess the sustenance of the cellular constituents of organoids over an extended period of culture using light microscopy only; i.e., 1 μm-thick sections stained with toluidine blue were evaluated. As cell death and salvage by autophagy are important considerations in mammalian cell cultures, particular attention was paid to morphological features including integrity of plasma membrane and nucleus along with disruptive features such as cell swelling, vacuolation of the endoplasmic reticulum, clumping of degraded ribosomes, pyknosis, karyorrhexis, and karyolysis.

### 4.7. Radioimmunoassay (RIA)

Spent medium was removed every 2 to 3 days from the cell cultures, pooled from multiple wells of the same passage and days, and stored at −80 °C. Samples were assessed with an equine luteinizing hormone (eLH) radioimmunoassay (RIA), which has been demonstrated to be an accurate tool for eCG quantification [42,43]. Assays were conducted by the CSU Endocrinology Laboratory as previously described [42], using LH antibody R15 and standard eLH-CSU-S8-GLP. Briefly, diluted samples were incubated with 200 μL rabbit anti-ovine LH (1:40,000 dilution) at 4 °C overnight. Then 100 μL ^125^I-oLH were added to each tube, and incubation was continued for an additional 24 h at 4 °C. A goat anti-rabbit IgG (200 μL, 1:100) was added to each tube to separate antibody bound from free hormone. This reaction was for 72 h at 4 °C. Subsequent to this incubation, 3 mL ice-cold PBS was added to each tube, and tubes were centrifuged for 30 min at 2800× *g*. Supernatants were decanted and radioactivity in the pellets was quantified by gamma spectroscopy. Multiple dilutions of each pool of culture medium was analyzed to make certain that the concentration of eCG in the samples was calculated using the most precise portion of the standard curve. Four RIAs were performed due to timing of sample collection with an inter-assay coefficient of variation of 19.5% and intra-assay coefficients of variation of 3.03%, 3.94%, 7.61%, and 14.89%. The lower limit of quantitation for the assays was 3.73 ± 0.49 ng/mL (mean ± SEM).

## Figures and Tables

**Table 1:** Experimental design.

Trial	Cell Isolation	Culture Method	Culture Medium	Incubator Settings
1	Enzymatic digestion	Organoid	Human trophoblast organoid medium [30]	37 °C, 5% CO_2_
2a	Enzymatic digestion	Organoid	Human trophoblast organoid medium [30]	37 °C, 5% CO_2_
2b	Mechanical isolation	Organoid	Human trophoblast organoid medium [30]	37 °C, 8% CO_2_
2c	Mechanical isolation	Monolayer	Equine chorionic girdle monolayer medium [34]	37 °C, 8% CO_2_

## Data Availability

The data presented in this study are available within the article.
